# Antimicrobial Effect of Clove Against Foodborne Pathogens in Ground Buffalo Meat During Refrigerated Storage

**DOI:** 10.3390/foods15010113

**Published:** 2025-12-31

**Authors:** Rawan Mohammed Saadeldeen, Amira Ibrahim Zakaria, Mirela Imre, Kálmán Imre, Samir Mohammed Abd-Elghany, Khalid Ibrahim Sallam

**Affiliations:** 1 Department of Food Hygiene, Safety, and Technology, Faculty of Veterinary Medicine, Mansoura University, Mansoura 35516, Egypt; redflouer@hotmail.com (R.M.S.); amera.zakaria@yahoo.com (A.I.Z.); drsamir@mans.edu.eg (S.M.A.-E.); 2 Clinical Education Department II, Faculty of Veterinary Medicine, University of Life Sciences “King Mihai I” from Timișoara, 300645 Timișoara, Romania; 3 Department of Animal Production and Veterinary Public Health, Faculty of Veterinary Medicine, University of Life Sciences “King Mihai I” from Timișoara, 300645 Timișoara, Romania; kalmanimre@usvt.ro

**Keywords:** natural additives, clove, foodborne pathogens, buffalo meatballs, shelf life, sensory attributes

## Abstract

Ground meat is highly perishable and has a short shelf life due to microbial contamination with food spoilage bacteria along with foodborne pathogens, which increases the risk of food poisoning. Controlling microbial growth by using chemical or synthetic food additives or preservatives is of great health concern. Natural, plant-derived antimicrobial food additives are safer alternatives. Therefore, the main objective of this study was to evaluate the antimicrobial efficacy of different forms and concentrations of clove against food spoilage and foodborne pathogens and to determine their ability to enhance sensory quality and extend the shelf life of buffalo meatballs during refrigerated storage. Clove oil (0.25, 0.50, and 1.0 g/kg), clove extract (0.5, 1.0, and 1.5 g/kg), and clove powder (2.5, 5.0, and 7.5 g/kg) were assessed against aerobic plate counts (APCs), psychotropic counts (PCs), and foodborne pathogens such as *Staphylococcus aureus*, *Salmonella enterica* serovar Typhimurium, and *Escherichia coli* O157:H7, artificially inoculated in buffalo meatballs. Clove oil, clove extract, and clove powder treatments showed a significant (*p* < 0.01) reduction in the counts of *S*. *aureus*, *S. enterica* serovar Typhimurium, and *E. coli* O157:H7 compared to control samples. Among all tested forms and concentrations of clove, clove oil at 1.0 g/kg proved to be the most effective against the tested pathogens, as by the end of storage (day 12), it achieved 5.3 and 5.56 log reductions in *S. aureus* and *S. enterica* serovar Typhimurium, respectively, along with complete reduction in *E. coli* O157:H7, followed by clove extract at 1.5 g/kg, which produced 4.2, 4.92, and 7.01 log reductions in the corresponding three foodborne pathogens. The results showed that different concentrations of clove oil and extract treatments applied effectively improved the sensory attributes (flavor, tenderness, juiciness, and overall acceptability) of buffalo meatballs, while the sensory properties of clove powder were considered unacceptable, as it alters the taste and smell of meat. The ground buffalo meat treated with different concentrations of clove oil, clove extract, and clove powder significantly reduced the growth of APCs and PCs during refrigerated storage, resulting in 1.5 to 2.6 log reductions with a prolonged shelf life ranging from 9 to 12 days. Overall effects on shelf life and meat quality showed that all clove forms significantly slowed microbial growth and extended the shelf life of buffalo meatballs to 9–12 days, in contrast to 6 days or less for the control. The findings indicate that clove oil and clove extract are promising natural preservatives capable of improving microbial safety, maintaining sensory attributes, and enhancing the overall quality of buffalo meatballs during refrigerated storage.

## 1. Introduction

The production of Buffalo meat in Egypt has remained relatively stable between 2019 and 2023. The production was 217,000 metric tons in 2019 and 210,859.35 metric tons in 2023 (https://www.tridge.com/tridge-woods/buffalo-meat/EG, accessed on 21 December 2025). Red meat constitutes an essential part of the human diet, providing a large variety of macro- and micronutrients, including good-quality proteins, essential amino acids, minerals, and vitamin B groups. Buffalo meat is a key protein source in many regions (Asia, the Middle East, Africa). Buffalo meat differs substantially from beef, poultry, and other red meats in terms of fat composition, pH, water-binding capacity, and microbial ecology, which may influence antimicrobial behavior. Ground meat products, such as burgers, meat patties, and meatballs, are widely consumed worldwide but are very susceptible to microbial contamination due to improper handling, preparation, or storage [1]. Due to the high moisture content and nutritional value of meat, it offers the perfect conditions for the growth of spoilage bacteria and foodborne pathogens such as *Staphylococcus aureus*, *Salmonella enterica*, and *Escherichia coli*.

Recently, the WHO estimated that foodborne pathogens are responsible for six hundred million cases of illness and approximately 420,000 deaths worldwide each year (https://www.who.int/activities/estimating-the-burden-of-foodborne-diseases, accessed on 21 December 2025). In developing countries, meat and poultry products are implicated in about 50% of foodborne illnesses annually [2].

The presence of *S*. *aureus* indicates poor worker cleanliness and inappropriate behaviors, such as coughing or sneezing near food, along with the failure to conduct cleaning and sanitizing operations for utensils and equipment in a manner that protects against contamination of food and food-contact surfaces [3,4]. In contrast, the existence of *E*. *coli* indicates fecal contamination of food [5]. Staphylococcal food poisoning symptoms typically appear suddenly, with intense nausea and vomiting occurring 30 min to 8 h after the contaminated food is consumed. Human *S*. *enterica* is one of the most common food-borne pathogens that causes several public health illnesses, including fever, diarrhea, and abdominal cramps. *E. coli* O157:H7 causes severe abdominal cramps, watery or bloody diarrhea, nausea, and vomiting, and in some cases may progress to hemolytic uremic syndrome (HUS), leading to kidney complications [6].

Microbial growth in meat and its products leads to the deterioration of flavor, color, texture, nutritional quality, and shelf life during storage. Controlling microbial growth by using food additives, which have antimicrobial properties, is a promising method for producing safe meat. Food additives are either natural or chemical substances added to enhance the shelf life of food, improve its appearance, and inhibit the growth of spoilage or pathogenic microorganisms. Many chemical additives are toxic and can indirectly impact consumer health; therefore, most studies have focused on natural additives derived from plants as safer alternatives to chemical additives, primarily due to the health risks associated with the latter [7].

Clove (*Syzygium aromaticum*) is a plant species of the *Myrtaceae* family, traditionally used as an analgesic, particularly for dental pain and other disorders, or as a food flavoring spice. This plant is rich in eugenol, gallic acid, and eugenol acetate and has potential benefits for cosmetic, pharmaceutical, agricultural, and food applications [8]. Clove is applied commercially as a spice or condiment to prolong the shelf life of food products by delaying spoilage [9,10]. The FDA classifies clove oil as safe for use as a food additive. Clove essential oil has antimicrobial, antiviral, antifungal, antioxidant, antihistamine, anti-inflammatory, and anticancer properties, with useful effects on the cardiovascular and immune systems [8].

Traditional food spices like clove and cinnamon are frequently employed in the food industry due to their unique scents and antibacterial and antifungal properties [11]. The antimicrobial activity of clove essential oil against Gram-positive and Gram-negative bacteria, for instance, *S. aureus*, *E. coli*, *Pseudomonas aeruginosa*, yeast, and mold, is attributed to eugenol, which is present in quantities up to 85% of the aromatic oil extract from cloves [12]. Eugenol could inhibit the synthesis of proteins and DNA by destroying microorganisms’ cell walls and membranes and permeating the cytoplasmic membranes [13]. The antiviral, antifungal, antibacterial, and anticarcinogenic qualities of spice plants have been validated by numerous investigations in recent years. Among the various spices, cloves have garnered the most attention because of their strong antibacterial and antioxidant properties [14].

Despite these advantages, the meat industry continues to face major challenges related to rapid spoilage, microbial contamination, and the short shelf life of processed meat products. Synthetic preservatives are often used to control microbial growth; however, their potential toxicological risks, such as carcinogenicity and adverse health effects, have raised significant consumer concern. This growing demand for clean-label and naturally preserved foods has intensified interest in plant-derived antimicrobials as safer alternatives. Clove, with its potent antimicrobial and antioxidant profile, represents a promising candidate for replacing or reducing chemical additives in meat systems. Although the antimicrobial properties of clove are well documented, a direct comparison of its oil, extract, and powder forms on the shelf-life and safety of ground buffalo meat is still lacking. Therefore, the present study was carried out to evaluate the effect of adding different concentrations of clove oil, clove extract, and clove powder as safe natural additives on the sensory attributes, microbial growth, and foodborne pathogens in ground buffalo meat during refrigerated storage.

## 2. Materials and Methods

### 2.1. Preparation of Sample for Sensory Evaluations and Shelf Life Determination

The experiment of sensory and shelf life determination of ground buffalo meat was completed on three independent occasions at different times, during which triplicate samples from both the control and clove-treated ground buffalo samples were examined on each occasion to explore the effect of added clove on the microbial population and sensory characteristics of ground buffalo meat. On each occasion, thirty kilograms of thigh meat with its subcutaneous fat from buffalo carcasses (slaughtered between 18 and 24 months of age and weighing 450 to 500 kg) were purchased from a regional butcher shop in Mansoura City, Egypt, packaged and conveyed in an icebox to the Food Hygiene, Safety, and Technology Department, Faculty of Veterinary Medicine, Mansoura University. The proximate compositional analysis of the buffalo meat used in this study showed moisture, protein, fat, and ash contents of 75 ± 2%, 22 ± 2%, 7.5 ± 1.5%, and 1.1 ± 0.1%, respectively. Clove powder was purchased from local distributors (Abu Auf, New Cairo, Egypt); clove extract was purchased from local distributors (Kenouz, Alexandria-Cairo Road, Egypt); while clove oil was obtained from a Local Pure Essential Oils & Herbs Co. (Purity; Kom Abo Radi industrial zone, Cairo, Egypt). According to the manufacturer, the compositional analysis of clove oil, ethanolic clove extract, and clove powder is shown in Table 1.

Thigh meat from Buffalo carcasses was cut and ground using a meat grinder (Moulinex, Mayenne, France). The ground meat was divided into ten groups (about 3 kg each). Nine groups were treated and thoroughly mixed with various concentrations of clove oil (0.25, 0.50, and 1.0 g/kg), clove extract (0.5, 1.0, and 1.5 g/kg), and clove powder (2.5, 5, and 7.5 g/kg), while the tenth group functioned as a control with no clove forms added. The clove concentrations designated in this study were based on previous literature, as well as preliminary trials and sensory acceptability thresholds. The treated and control buffalo meat were shaped into approximately 30 g meatballs, put in polyethylene bags, tagged, and refrigerated at 4 °C for 12 days to evaluate their sensory attributes and shelf life by estimating the total aerobic plate counts and psychotropic counts.

### 2.2. Sensory Evaluation

The experiment of sensory and shelf life evaluation of ground buffalo meat was done on three independent occasions at different times. Sensory evaluation for the ten groups of buffalo meatball samples was performed at 0, 3, 6, 9, and 12 days of storage. Briefly, buffalo meatballs from each group were put on a sanitized aluminum foil tray and roasted in an electric oven set to 180 °C for 20 min. Cooked meatball samples were randomly coded with 3-digit numbers to ensure impartiality and presented to twenty-five semi-trained panelists comprising staff members and postgraduate students from the Food Hygiene, Safety, and Technology Department, Faculty of Veterinary Medicine, Mansoura University, who were asked to evaluate the clove flavor intensity, juiciness, tenderness, and overall acceptability using an eight-point hedonic scale that went from 1 (stands for uncharacteristic beef flavor, tough, dry sample) to 8 (stands for extremely characteristic beef flavor, tender, juicy sample) [15]. The Beef Sensory Evaluation Form of Texas Tech University was adopted and submitted as a Supplementary Table (Table S1). One session was held on every evaluation occasion of the three independent trials on days 0, 3, 6, 9, and 12 (3 sessions in total at 3-day intervals with 25 panelists). To maintain objectivity, panelists were directed to wash their mouths and palate with warm water for 30 s between tasting each meatball sample.

The statement “I understand that my answers are confidential, and I consent to take part in this survey” was given by sensory evaluation participants as informed consent, whereby participation in the survey required a positive response. Participants may discontinue the survey at any time without giving a reason. The food products tested were considered safe to consume.

### 2.3. Shelf Life Estimation by Determination of Aerobic Plate Counts (APCs) and Psychotropic Plate Counts (PPCs)

Buffalo meatball samples weighing 10 g were taken from the nine treated groups and the control group at day 0 and at each 3-day interval throughout the 12 days of the storage period. Ninety milliliters of 0.1% peptone water were used to homogenize the samples (Plate Number 1; Oxoid Ltd., Basingstoke, UK) using a blender (Moulinex, Mayenne, France). In 20 mL test tubes, tenfold serial dilutions were prepared up to 10^−6^ by mixing 1 mL of the homogenized meat sample with 9 mL of peptone water solution. The appropriate dilutions were selected, and 0.1 mL aliquots were inoculated and spread evenly over the surface of each duplicate count agar plate (CM0325, Oxoid Ltd., Basingstoke, UK) [16]. The plates were inverted and incubated at 30 °C for 72 h to enumerate APCs and at 7 °C for 10 days to determine PPCs [17]. Bacterial colonies were counted and recorded for the buffalo meatball samples as log_10_ CFU/g.

### 2.4. Effect of Clove Oil, Extract, and Powder on Food Poisoning Bacteria

#### 2.4.1. Pathogenic Strains and Inoculum Preparation

The bacterial strains used in this study, including *E*. *coli* O157:H7, *S*. *aureus* (MRSA), and *S*. *enterica* serovar Typhimurium, were formerly recovered from meat and maintained in the culture collection of our laboratory. The identity of each strain was confirmed by conventional biochemical tests followed by PCR targeting specific virulence or species-confirmatory genes. A single colony from each specific bacterial pathogen was incubated in a sterile nutrient broth of 100 mL (Oxoid CM0001B; Oxoid Ltd., Basingstoke, UK) at 37 °C for 24 h. A spectrophotometer was utilized to adjust the bacterial culture concentrations to an optical density of 0.5 at 625 nm to produce the inoculum with an estimated count of 4 × 10^8^ CFU/mL (about 8.6 log_10_ CFU/mL) according to the McFarland turbidity standard of 0.5. All microbiological procedures were conducted under Biosafety Level 2 (BSL-2) conditions, using a Class II biosafety cabinet (Shanghai Yantai Science & Technology Co., Ltd., Yantai, Shandong, China) and following institutional safety protocols. Contaminated materials were autoclaved before disposal

#### 2.4.2. Inoculation of Clove-Treated Ground Buffalo Meat with Foodborne Pathogens

The experiment determining the antimicrobial effect of different concentrations of various forms of clove powder on the foodborne pathogens inoculated into ground buffalo meat was also done on three independent occasions at varying times, during which triplicate samples from each of the control and the clove-treated ground buffalo meat were tested. On each occasion, ten kilograms of fresh buffalo meat were bought from the same butcher shop, ground, and categorized into 10 groups, each weighing 1 kg, to assess the antibacterial effect of clove in three different forms against *S*. *enterica*, *E*. *coli* O157:H7, and *S*. *aureus*. The nine groups were treated by mixing thoroughly with varying concentrations of clove oil (0.25, 0.50, 1.0 g/kg), clove extract (0.5, 1.0, and 1.5 g/kg), or clove powder (2.5, 5, 7.5 g/kg). The tenth group served as a control. Each treated group, along with the control group, was further subdivided into 4 subgroups, each weighing 250 g. These subgroups were inoculated with either *S*. *aureus*, *S*. *enterica* serovar Typhimurium, or *E*. *coli* O157:H7. A quantity of 2.5 mL from each prepared bacterial sample (approximately 8.6 log_10_ CFU/mL) was cautiously and thoroughly mixed with each meat group (250 g) (2.5 × 8.6 log CFU/250) for about 2 min by hand while wearing sterile gloves to generate a homogenous paste with about 6.6 log_10_ CFU/g of the particular food-poisoning organism. The meatballs were then formed manually (25–30 g), packed in polyethylene bags, labeled, and stored at 3 °C under aerobic conditions for 12 days.

#### 2.4.3. Recovery of the Inoculated Specific Food Poisoning Bacteria

The inoculated pathogens were then recovered from the inoculated meat using the conventional spread plate technique in the appropriate selective media. A volume of 0.1 mL aliquot from the appropriate dilution was spread onto Xylose Lysine Desoxycholate agar (Oxoid CM0469; Oxoid Ltd., Basingstoke, UK) for recovery of *Salmonella enterica* serovar Typhimurium, Sorbitol MacConkey Agar (Oxoid CM0813; Oxoid Ltd., Basingstoke, UK) supplemented with potassium tellurite and cefixime (Oxoid SR0172E; Oxoid Ltd., Basingstoke, UK) for recovery of *E. coli* O157:H7, and Baird Parker selective agar (Oxoid CM275; Oxoid Ltd., Basingstoke, UK) with egg-yolk tellurite emulsion for recovery of MRSA. The selective media plates were incubated for 24 h at 37 °C, and subsequently, the numbers of specific colonies for each bacterium were enumerated and expressed as log_10_ CFU/mL. Typical colonies grown on selective media were further verified by standard biochemical tests and PCR assays targeting the *nuc*, *invA*, and *rfbO*_157_ marker genes for the confirmation of *S. aureus*, *S. enterica*, and *Escherichia coli* O157:H7, respectively [18,19,20].

### 2.5. Statistical Analysis

Statistical data analysis was conducted using SPSS Statistics 27.0 for Windows (SPSS Inc., Chicago, IL, USA) and presented as mean ± SE for all values across the three separate experimental occasions. The study employed a completely randomized design with four groups: nine treatments (ground buffalo with different concentrations of clove oil, extract, and powder, alongside the tested pathogen) and a control (ground buffalo with 0% clove oil, extract, or powder). Each treatment (control, clove oil, clove extract, clove powder at different concentrations) was prepared in three independent biological replicates. For each biological replicate, microbiological analyses (foodborne pathogen counts, spoilage bacteria counts) and sensory attributes evaluation were performed in triplicate as technical replicates.

To determine significant differences during storage periods of 0, 3, 6, 9, and 12 days, the Generalized Linear Mixed Models (GLMM) approach was used to analyze the data for the dependent variables. The model included treatments, storage duration, and their interaction as fixed effects. Treatments and storage days were designated as fixed variables, while replications served as a random effect in the statistical design. The mean values were compared using the Tukey multiple comparison test to identify any significant differences. Differences were considered significant when *p* < 0.01. Results are expressed as mean ± SE.

**Figure 1:**
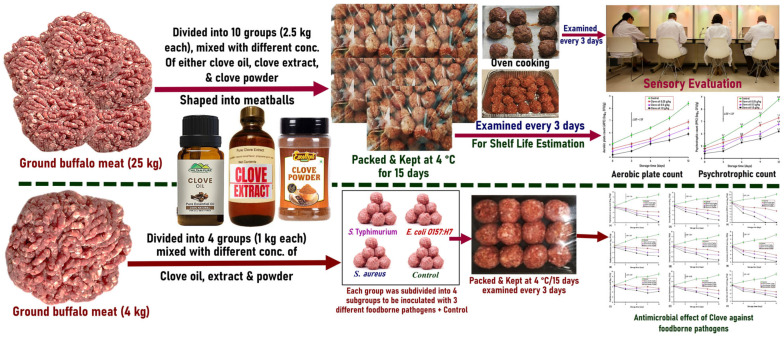
Graphical summary of the experimental design of the present study.

GLMM was used to evaluate sensory evaluation data and test for differences in sensory qualities between the various clove addition concentrations. In addition to the clove form concentrations (fixed effect), the panelists and the sessions (random effect) were the explanatory factors in each GLMM. The Post Hoc Least Squares Differences (LSD) test was used to compare the mean changes among the different treatments. The mean value differences were considered significant at *p* < 0.05. A graphical summary of the experimental design and of this study is shown in Figure 1.

## 3. Results and Discussion

### 3.1. Sensory Attributes and Consumer Acceptance

**Figure 2:**
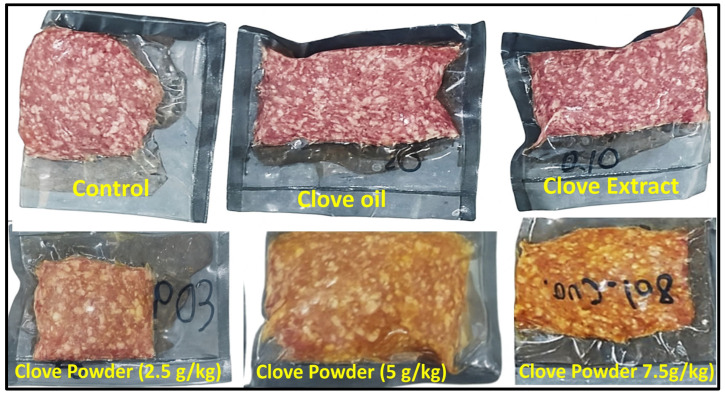
Visual comparison of ground buffalo meat illustrating the effects of incorporating clove oil, clove extract, and clove powder at different concentrations on meat color. Treatments containing clove oil and clove extract show no noticeable color alteration relative to the control, whereas increasing levels of clove powder produce a dose-dependent darkening of the meat surface.

Interestingly, the incorporation of clove oil or clove extract at all tested concentrations did not produce any noticeable alterations in the color attributes of ground buffalo meat compared with the control samples. Such stability may be attributed to the clear, pigment-free nature of these forms, which disperse uniformly within the meat matrix without contributing visible chromatic compounds. In contrast, clove powder produced a noticeable and dose-dependent darkening of the meat. This effect is likely due to the presence of natural brown pigments, fiber particles, and non-soluble phenolic constituents in the powdered form, which become increasingly apparent as the concentration increases. The particulate nature of clove powder also limits its uniform distribution, enhancing the visual impact of its inherent color. These compositional differences among clove forms explain the observed variation in color modification (Figure 2).

**Figure 3:**
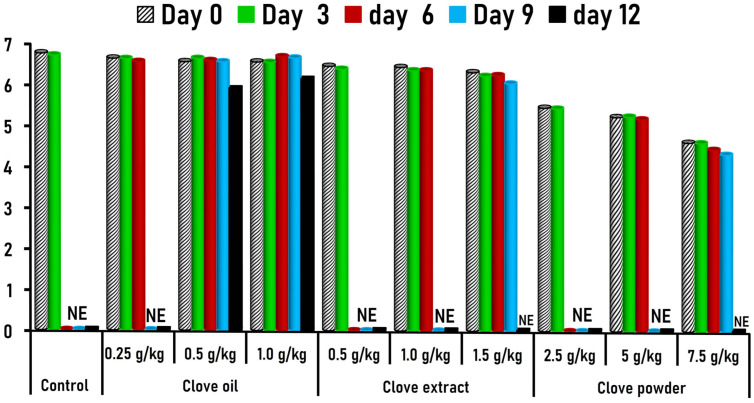
Overall acceptability (overall mouthfeel) scores of buffalo meatballs incorporated with different clove forms at various concentrations during a storage period of 12 days. NE: not evaluated due to obvious deteriorative changes in the product.

The sensory attributes of control and ground buffalo meat treated with various levels of clove oil, clove extract, and clove powder during 12 days of cooled storage are clarified in Table 2. Buffalo meatballs handled with varying concentrations of clove powder indicated a significant (*p* < 0.05) difference in clove flavor intensity, and they showed the lowest acceptable scores compared to both control and other clove-treated samples (Table 2). The potent flavor of clove comes from aromatic compounds like eugenol, eugenol acetate, β-caryophyllene, and methyl salicylate, which are heat-stable. Furthermore, no significant changes were observed in the distinctive buffalo meat flavor, juiciness, tenderness, or overall acceptability between the control meatballs and those treated with clove oil and clove extract at different concentrations throughout the 12-day storage period (Table 2). The decline in overall acceptability (overall mouth feeling) scores over storage time is shown in Figure 3.

Comparable findings were reported in India by Sharma et al., who observed that the overall acceptability scores for control and fresh chicken sausages treated by 0.25% clove oil were comparable [21]. Likewise, Zhang et al. demonstrated nonsignificant variances (*p* > 0.05) in flavor and texture scores between the control and Chinese-style sausages treated with clove extracts [22]. Additionally, Ahmed and Mohammed reported that the polyphenolic compounds found in cloves significantly enhance the tenderness of beef patties stored at 4 °C for 10 days [23]. The sensory evaluation in the present study was not conducted for the control meatballs, and the samples treated with clove extract (0.5 g/kg) and clove powder (2.5 g/kg) were excluded from evaluation starting from day 6 and thereafter due to noticeable deteriorative changes in the ground meat. On day 9, however, buffalo meatballs treated with clove oil (0.25 g/kg), clove extract (1.0 g/kg), and clove powder (5 g/kg) showed deteriorative changes. In comparison, meatballs treated with clove extract (1.5 g/kg) and clove powder (7.5 g/kg) exhibited signs of deterioration on day 12 of storage (Table 2). Interestingly, buffalo meatballs treated with clove oil at 0.50 and 1.0 g/kg showed no deteriorative changes in odor or color up to the final day of storage (day 12) and were deemed acceptable by the panelists. Our sensory evaluation revealed that the addition of clove oil (0.25, 0.50, 1.0 g/kg) and clove extract (1.0 and 1.5 g/kg) can be utilized as natural food additives, as they resulted in acceptable sensory scores and prolonged the shelf life of treated buffalo meatballs by 3 to 6 days compared to the control samples.

The improvement in flavor, juiciness, and overall acceptability in samples treated with clove oil and extract may be related to their antioxidant properties, which delay lipid oxidation and protein degradation, thereby preserving meat quality during refrigerated storage. Conversely, clove powder adversely affected sensory attributes due to its higher load of raw aromatic compounds and coarse texture, which imparted a strong, undesirable taste and odor that masked the natural meat flavor. Together, these mechanisms explain why clove derivatives enhanced microbial safety and sensory quality to varying degrees across treatments.

### 3.2. Microbial Quality of Buffalo Meatballs Treated with Clove Oil, Extract, and Powder During Refrigerated Storage

#### 3.2.1. Effect of Clove Oil, Clove Extract, and Clove Powder on APCs

**Figure 4:**
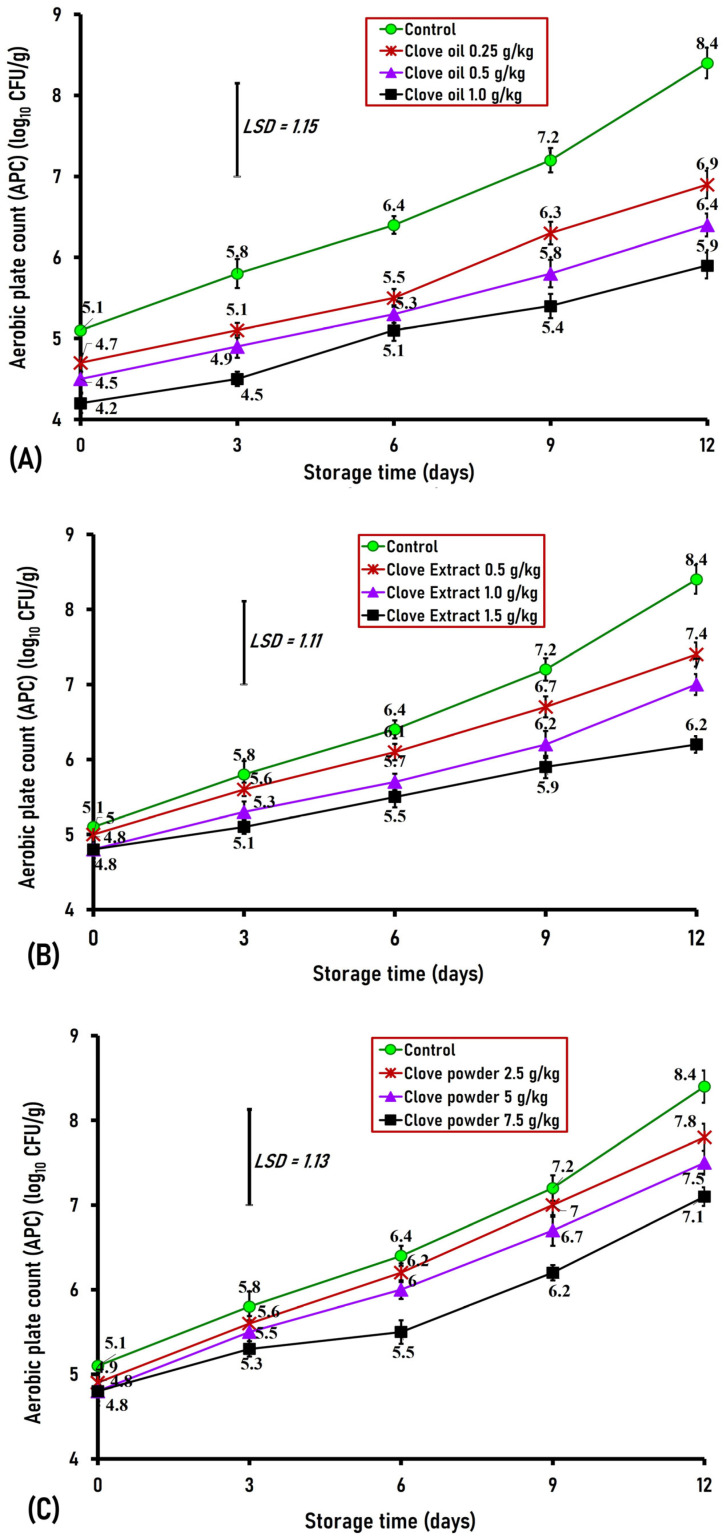
Aerobic plate counts (APC) in ground buffalo meat treated with different concentrations of clove oil (**A**), extract (**B**), and powder (**C**) during cold storage at 4 °C for 12 days. Data represent the mean ± SE values on each day of analysis. SE is represented by vertical bars; LSD was calculated at *p* < 0.01.

The aerobic plate counts are commonly used to evaluate the microbial load of fresh poultry and meat products, providing valuable information on their quality, safety, and shelf life [24]. Our results revealed that the APCs of control and clove-treated buffalo meatballs ranged from 4.2 to 5.1 log_10_ CFU/g on the first day of storage, with no noticeable variations (*p* < 0.05) in APCs among all clove-treated buffalo meatballs as well as the control samples (Figure 4A–C). On days three and six of storage, only ground buffalo meat treated with 1.0 g/kg of clove oil caused a noticeable decrease (*p* < 0.05) in APCs by 1.3 log in comparison to control samples for both days (Figure 4A–C). On the ninth day of storage, APCs declined (*p* < 0.01) in buffalo meatballs treated with 0.50 and 1.0 g/kg clove oil and 1.5 g/kg clove extract, resulting in a significant (*p* < 0.01) reduction in APCs by 1.4, 1.8, and 1.3 logs, respectively, in contrast to the original samples. On the last day (day 12) of storage, ground buffalo meat treated with clove oil (0.25, 0.50, and 1.0 g/kg), clove extract (1.0, and 1.5 g/kg), and clove powder (7.5 g/kg) induced a significantly lower (*p* < 0.01) APCs by 1.5, 2, 2.5, 1.4, 2.2, and 1.3 logs, respectively, in contrast to the original group (6.9, 6.4, 5.9, 7, 6.2, and 7.1, respectively, contrasted with 8.4 log_10_ CFU/g) (Figure 4A–C).

The maximum allowable limit for APCs is 7.0 log_10_ CFU/g as indicated by the ICMSF [25]. In our study, the APCs of the control samples exceeded this limit during the ninth day of storage. In contrast, ground buffalo meat containing clove extract (0.5 and 1.0 g/kg) and clove powder (2.5, 5, and 7.5 g/kg) reached the maximal allowable limit by day 12 (Figure 4B,C). Interestingly, the APCs of buffalo meatballs treated with clove oil (0.25, 0.50, and 1.0 g/kg) and clove extract (1.5 g/kg) were consistently below the 7 log_10_ CFU/g acceptable limit throughout the 12 days of storage (Figure 4A,B).

The antimicrobial activity of clove is attributed to multiple components, including eugenol, eugenol acetate, beta-caryophyllene, rhamnocitrin, oleanolic acid, and gallic acid [26]. Previous studies have documented the antimicrobial effect of clove in different meat products. For instance, Sharma et al. found a significant decline in total plate counts of fresh chicken sausage samples treated with clove essential oils at concentrations of 0.125%, 0.25%, 0.5%, and 1% [27]. Also, Ali et al. indicated that beef sausage treated with clove powder (0.5%) and clove extract (0.5%) exhibited a significant decrease in mesophilic bacterial counts by day 6 of storage, with counts of 5.69 and 5.30 log_10_ CFU/g, respectively, compared to 6.85 log_10_ CFU/g in the control sample [28]. Furthermore, methanol and ethanol extracts of cloves demonstrated a potent antimicrobial effect on four Gram-positive and four Gram-negative bacteria in chicken meat broth [29].

#### 3.2.2. Effect of Clove Oil, Clove Extract, and Clove Powder on PPCs

**Figure 5:**
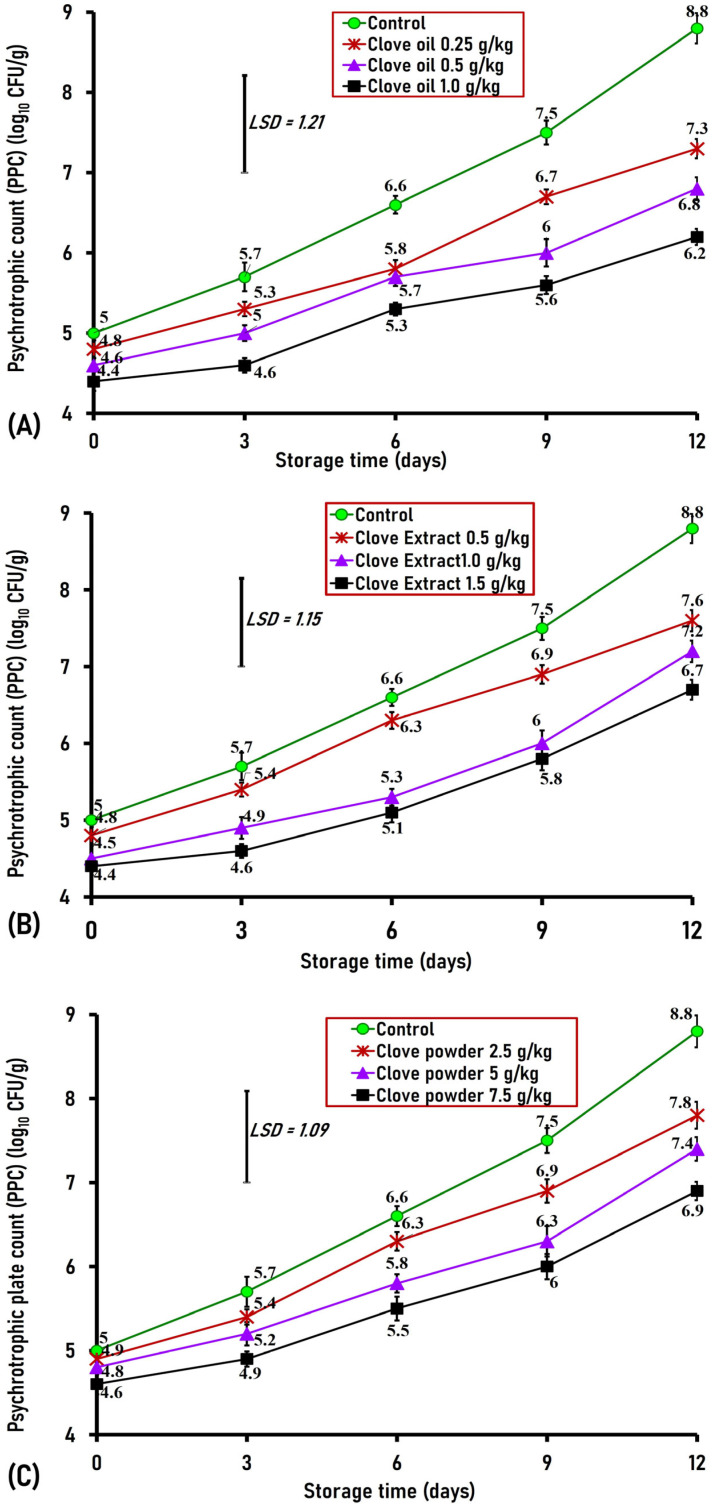
Psychrotrophic plate counts (PPC) in ground buffalo meat treated with different concentrations of clove oil (**A**), extract (**B**), and powder (**C**) added during cold storage at 4 °C for 12 days. Data represent the mean ± SE values on each day of analysis. SE is represented by vertical bars; LSD was calculated at *p* < 0.01.

Bacteria that develop on chilled meat are regarded as psychrotrophs. These bacteria include both Gram-negative genera, such as *Enterobacteriaceae* and *Pseudomonas* spp., and Gram-positive types, such as lactic acid bacteria [30]. Our findings showed that the PPCs on the first day of storage ranged from 4.4 log_10_ CFU/g in ground buffalo meat samples preserved with clove oil at a 1.0 g/kg concentration to 5.0 log_10_ CFU/g in the untreated original samples. No discernible variations in PPCs were found between the control and samples treated with clove oil, clove extract, and clove powder (Figure 5A–C). By the third day, the PPC of ground buffalo meat treated with 1 g/kg of clove oil was 1.1 logs lower (*p* > 0.1) than that of the control (4.6 vs. 5.7 log_10_ CFU/g). By day six, the PPCs of ground buffalo meat treated with 1.0 g/kg of clove oil, 1.0 and 1.5 g/kg of clove extract, and 7.5 g/kg of clove powder were significantly lower than those of the untreated sample by 1.3, 1.3, 1.5, and 1.1 logs, respectively, (5.3, 5.3, 5.1, and 5.5 versus 6.6 log_10_ CFU/g in the control) (Figure 5A–C). On day 9 of storage, clove oil (0.5 and 1.0 g/kg), clove extract (1.0 and 1.5 g/kg), and clove powder (5 and 7.5 g/kg) exhibited a significant (*p* < 0.01) decrease in PPCs by 1.5, 1.9, 1.5, 1.7, 1.2, and 1.5 logs, respectively, compared to the untreated sample (6.0, 5.6, 6, 5.8, 6.3, and 6.0 versus 7.5 log_10_ CFU/g in the control) (Figure 5A–C). On the final day of storage, the untreated control samples displayed a high psychrotrophic count of 8.8 log_10_ CFU/g; in contrast, all clove-treated samples observed significantly (*p* < 0.01) lower PPCs than the control, except the sample treated with 2.5 g/kg of clove powder. The greatest decrease of 2.6 logs was observed in buffalo meatballs treated with clove oil at 1.0 g/kg (6.2 vs. 8.8 log_10_ CFU/g in the control), followed by decreases of 2.1 and 2 logs in 1 g clove extract- and 0.5 g clove oil-treated samples, respectively. The inhibitory effect of clove oil against psychrotrophic bacteria is attributed to eugenol, a major component that disrupts the cytoplasmic membrane of microorganisms, resulting in increased cellular permeability and cell lysis [31].

The addition of clove oil (0.25, 0.50, and 1.0 g/kg), clove extract (0.5, 1.0, and 1.5 g/kg), and clove powder (5 and 7.5 g/kg) to ground buffalo meat effectively (*p* < 0.01) inhibited psychrotrophic bacterial growth. Hence, these three forms of clove can be safely utilized as natural preservative to enhance the meat quality and extend its shelf life of during cold storage.

An in vitro study revealed that clove essential oil at 0.16% and 0.08% resulted in potent antibacterial activity against *Pseudomonas aeruginosa* [32]. Also, clove essential oil significantly reduced the growth of psychotrophic bacterial populations in raw buffalo patties during refrigerated storage [33]. Additionally, clove powder exhibited strong inhibitory effects in vitro against *Pseudomonas fluorescence*, with minimum inhibitory concentrations of 0.2% *w*/*v* as the powder concentration increased from 0.5% to 2.5% *w*/*v* [34]. Furthermore, Keskin and Toroglu examined the antibacterial effects of extracts prepared from 12 spices against *P. aeruginosa* in vitro, and they indicated that clove extract produced the strongest inhibitory effects compared to the other spice extracts [35].

The shelf-life extensions obtained in clove-treated meatballs can be directly linked to the point at which microbial counts surpassed accepted spoilage thresholds and when sensory acceptability declined. In fresh meat products, aerobic plate counts (APCs) reaching 6–7 log CFU/g and psychrotrophic counts (PCs) exceeding 7 log CFU/g are widely regarded as the upper microbiological limits defining the end of shelf life. In the control samples, APCs and PCs exceeded these limits by day 6, which corresponded to clear sensory deterioration and therefore established the control’s maximum shelf life. In contrast, clove oil and clove extract treatments suppressed microbial growth sufficiently to keep APCs and PCs below 7 log CFU/g until days 9–12, depending on concentration. This delayed microbial proliferation was consistent with the maintenance of acceptable flavor, tenderness, juiciness, and overall acceptability during the same period. Clove oil and clove extract at different concentrations demonstrated a significant antimicrobial effect, with microbial loads remaining below spoilage thresholds through day 12, which matched the point at which sensory quality also remained within acceptable limits. Although clove powder treatments also delayed microbial growth to below spoilage thresholds until approximately day 9, their sensory scores declined earlier due to the powder’s strong flavor and visible color impact, thereby limiting shelf life based on sensory rejection rather than microbial failure. Collectively, these findings demonstrate that the shelf-life extension of 9–12 days in clove-treated meatballs results from the combined effect of microbial suppression and the ability of each clove form to preserve sensory quality throughout storage.

While the present study focused on antimicrobial effectiveness, clove essential oil, extract, and powder are also recognized to exhibit antioxidant activity owing to their high phenolic content, predominantly eugenol. Future research should estimate the oxidative stability of meat products to determine whether clove-derived additives can simultaneously enhance microbial safety and reduce lipid oxidation during storage. Integrating antimicrobial and antioxidant assessments would provide a more comprehensive understanding of clove’s preservative potential in buffalo meat.

### 3.3. Effect of Clove Oil, Extract, and Powder on Foodborne Pathogens Artificially Inoculated into Ground Buffalo Meat During Refrigerated Storage

#### 3.3.1. Effect of Clove Oil, Extract, and Powder on Methicillin-Resistant *S. aureus* (MRSA)

**Figure 6:**
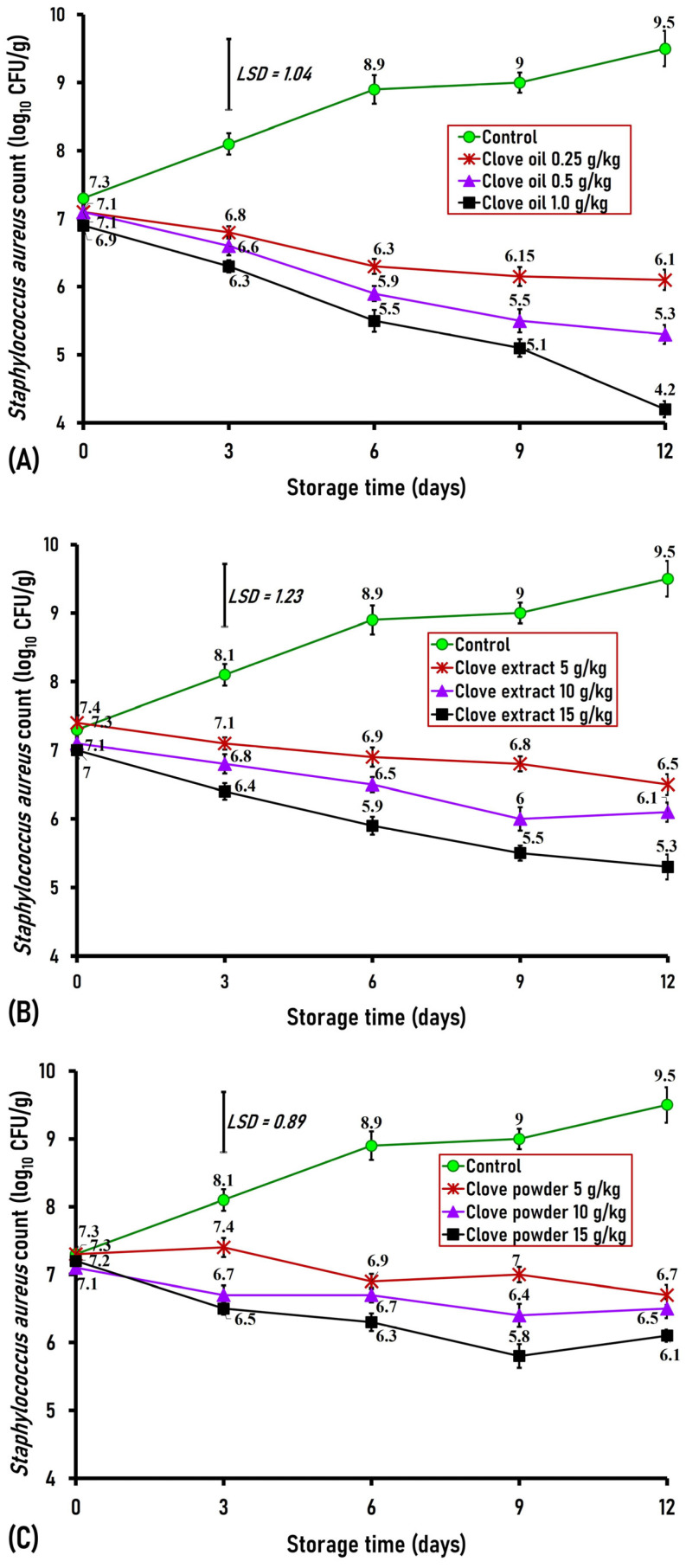
Antimicrobial effect of different concentrations of clove oil (**A**), extract (**B**), and powder (**C**) against methicillin-resistant *Staphylococcus aureus* (MRSA) artificially inoculated into ground buffalo meat stored at 4 °C for 12 days. Data represent the mean ± SE values on each day of analysis. SE is represented by vertical bars; LSD was calculated at *p* < 0.01.

Food handlers often contribute to food contamination with *S*. *aureus* due to direct contact with hand lesions or through coughing and sneezing [3,4]. In the present study, no discernible variation in the initial counts of inoculated MRSA on day 0 was observed between the control and clove-treated ground buffalo meat, which showed initial counts ranging between 6.9 and 7.3 log_10_ CFU/g (Figure 6A–C). On the third day, ground buffalo meat treated with clove oil (0.25, 0.50, and 1.0 g/kg), clove extract (1 and 1.5 g/kg), and clove powder (5 and 7.5 g/kg) revealed a significant decrease (*p* < 0.01) in the counts of *S*. *aureus* (MRSA) by 1.3, 1.5, and 1.8 logs for clove oil; 1.3 and 1.7 logs for clove extract; and 1.4 and 1.6 logs for clove powder, respectively, compared to the control count, which contained a high count of 8.1 log_10_ CFU/g (Figure 6A–C). On the sixth day of storage, ground buffalo meat treated with clove oil (0.25, 0.50, and 1.0 g/kg), clove extract (0.5, 1.0, and 1.5 g/kg), and clove powder (2.5, 5, and 7.5 g/kg) showed a significant (*p* < 0.01) decline in *S*. *aureus* count by 2.6, 3.0, and 3.4 logs for clove oil; 2, 2.4, and 3 logs for clove extract; and 2, 2.2, and 2.6 logs for clove powder, respectively, when compared to the control of 8.9 log_10_ CFU/g (Figure 6A–C). On the ninth day and thereafter, ground buffalo meat treated with clove oil (0.25, 0.5, and 1.0 g/kg), clove extract (0.5, 1, and 1.5 g/kg), and clove powder (2.5, 5, and 7.5 g/kg) exhibited a significant reduction (*p* < 0.01) in *S*. *aureus* counts by 2.8, 3.5, and 3.9 logs for clove oil-treated samples; 2.2, 3, and 3.5 logs for clove extract-treated samples; and 2, 2.2, and 3.2 logs for clove powder-treated samples, respectively, in contrast to the control sample of 9 log_10_ CFU/g (Figure 6A–C). At the end of the storage period (day 12), the control ground buffalo meat revealed a high count of 9.5 log_10_ CFU/g for *S*. *aureus* (MRSA), while ground buffalo meat mixed with clove oil (0.25, 0.50, and 1.0 g/kg), clove extract (0.5, 1.0, and 1.5 g/kg), and clove powder (2.5, 5, and 7.5 g/kg) showed significantly (*p* < 0.01) lower MRSA counts by 3.4, 4.2, and 5.3 logs for clove oil; 3, 3.4, and 4.2 logs for clove extract; and 2.8, 3, and 3.4 logs for clove powder, respectively (Figure 6A–C). Our findings indicate the strong antimicrobial effect of clove oil, clove extract, and clove powder as preservatives or food additives against *S*. *aureus*.

The approximately 2 log increase in *S*. *aureus* counts observed in the refrigerated control ground buffalo meat over 12 days is uncommon and may be attributed to several factors, including the high initial microbial load (7.3 log_10_ CFU/g), strain-dependent characteristics—particularly as the inoculated strain was originally isolated from chilled meat—and the influence of a nutrient-rich matrix, especially protein- and fat-rich foods, which may support persistence and growth under refrigeration.

The present finding concerning the impact of clove on *S*. *aureus* growth is closely consistent with that of Nassan et al., who indicated that clove extract exhibited strong inhibitory effects in vitro against *S*. *aureus*, with a minimum inhibitory concentration of 2 mg/mL [36]. Additionally, Kuang et al. demonstrated that clove powder exhibited potent antimicrobial activity in vitro against *S*. *aureus* [34]. In this context, Shan et al. revealed that phenolic compounds from various plants can inhibit several food-borne pathogens [37].

#### 3.3.2. Effect of Clove Oil, Extract, and Powder on *S. enterica* Serovar Typhimurium

**Figure 7:**
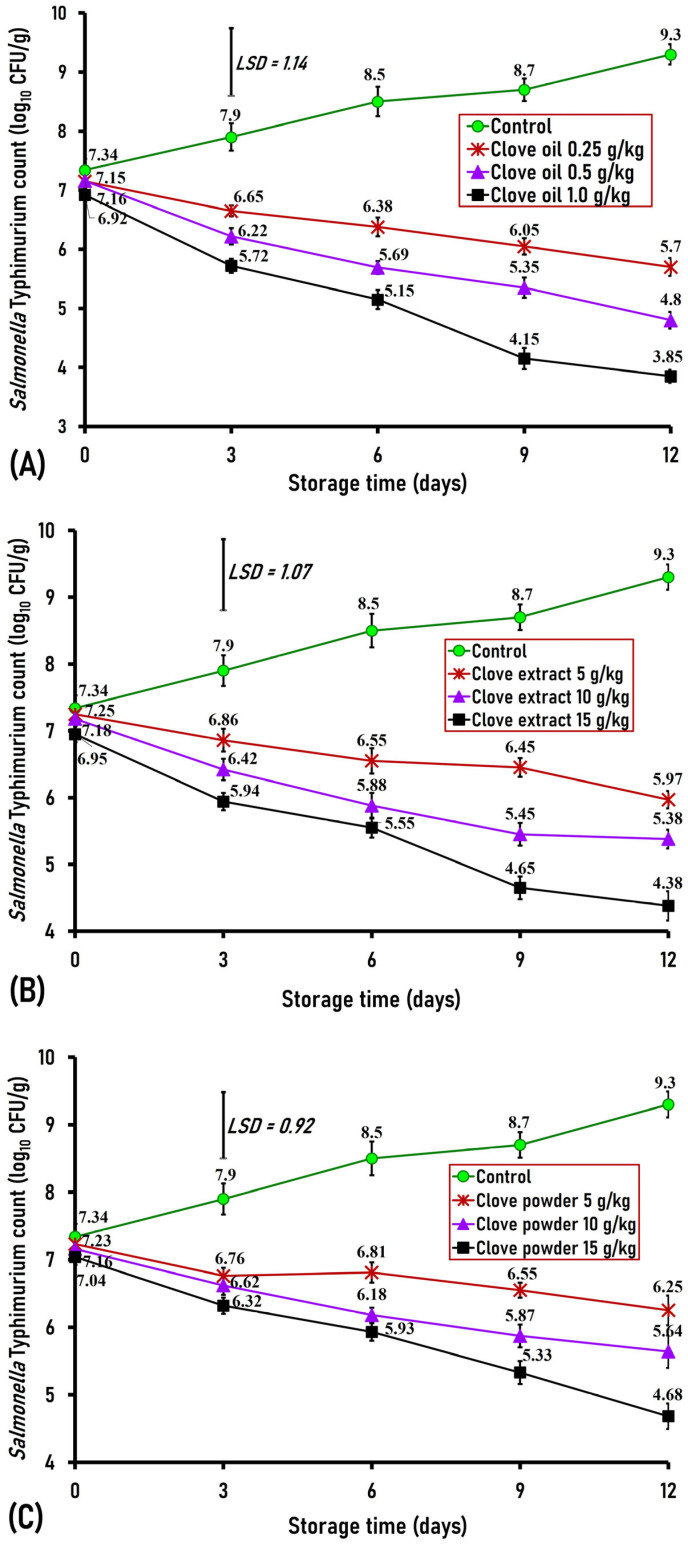
Antimicrobial effect of different concentrations of clove oil (**A**), extract (**B**), and powder (**C**) against *Salmonella enterica* serovars Typhimurium artificially inoculated into ground buffalo meat stored at 4 °C for 12 days. Data represent the mean ± SE values on each day of analysis. SE is represented by vertical bars; LSD was calculated at *p* < 0.01.

*Salmonella* is a major worldwide risk to public health, causing 93.8 million foodborne outbreaks and over 155,000 fatalities annually worldwide [38]. In the current study, the initial (day 0) *S*. *enterica* serovar Typhimurium counts artificially inoculated in both the control and clove-treated ground buffalo meat ranged from 6.92 to 7.34 log_10_ CFU/g, indicating no significant difference between the treated and control samples (Figure 7A–C). On the third day, however, buffalo meatballs treated with clove oil (0.25, 0.50, and 1.0 g/kg), clove extract (1.0, 1.5 g/kg), and clove powder (2.5, 5, and 7.5 g/kg) exhibited a significant (*p* < 0.01) decrease in *S*. *enterica* serovar Typhimurium counts by 1.25, 1.68, 2.18 logs for clove oil-treated samples; 1.48, 1.96 logs for clove extract-treated samples; and 1.14, 1.28, 1.58 logs for clove powder-treated samples, respectively, as opposed to the control (7.9 log_10_ CFU/g) (Figure 7A–C). On the sixth day of storage, ground buffalo meat treated with clove oil (0.25, 0.50, and 1.0 g/kg), clove extract (0.5, 1.0, and 1.5 g/kg), and clove powder (2.5, 5, and 7.5 g/kg) exhibited a significant (*p* < 0.01) decrease in *S. enterica* serovar Typhimurium counts by 2.12, 2.81, 3.35 logs for clove oil; 1.95, 2.62, 2.95 logs for clove extract; and 1.69, 2.32, 2.57 logs for clove powder, respectively, in contrast to the control (8.5 log_10_ CFU/g) (Figure 7A–C).

On the ninth day of storage, ground buffalo meat treated with clove oil (0.25, 0.50, and 1.0 g/kg), clove extract (0.5, 1.0, and 1.5 g/kg), and clove powder (2.5, 5, and 7.5 g/kg) demonstrated a significant (*p* < 0.01) decrease in *S*. *enterica* serovar Typhimurium counts by 2.65, 3.35, and 4.55 logs for clove oil; 2.25, 3.25, 4.05 logs for clove extract; and 2.15, 2.83, 3.37 logs for clove powder, respectively, in contrast to the control (8.7 log_10_ CFU/g) (Figure 7A–C). On the last day (day 12) of storage period, ground buffalo meat of the control revealed a high *S. enterica* serovar Typhimurium count of 9.3 log_10_ CFU/g, in contrast the buffalo meatballs treated with clove oil (0.25, 0.50, and 1.0 g/kg), clove extract (0.5, 1.0, and 1.5 g/kg) and clove powder (2.5, 5, and 7.5 g/kg) exhibited much lower counts by 3.6, 4.5, 5.45 logs for clove oil-treated samples; 3.33, 3.92, 4.92 logs for clove extract-treated samples; and 3.05, 3.66, 4.62 logs for clove powder-treated samples, respectively (Figure 7A–C).

The current results indicate that clove, in three forms at different concentrations, reveals potent antibacterial activity toward *S*. *enterica* serovar Typhimurium as a food additive. Similarly, Zengin and Baysal demonstrated that clove oil limited the growth of experimentally inoculated *S*. *enterica* serovar Typhimurium in ground beef [39]. Additionally, Angienda et al. tested the antimicrobial activity of four spices in vitro, and they indicated that clove oil showed the highest minimum inhibitory concentration of 2.50% *v*/*v* [40]. Furthermore, Shan et al. assessed the antimicrobial effects of five spice extracts and found that the raw pork samples treated with clove extract had the lowest counts of *S. enterica* [41].

#### 3.3.3. Effect of Clove Oil, Clove Extract, and Clove Powder on *Escherichia coli* O157:H7

**Figure 8:**
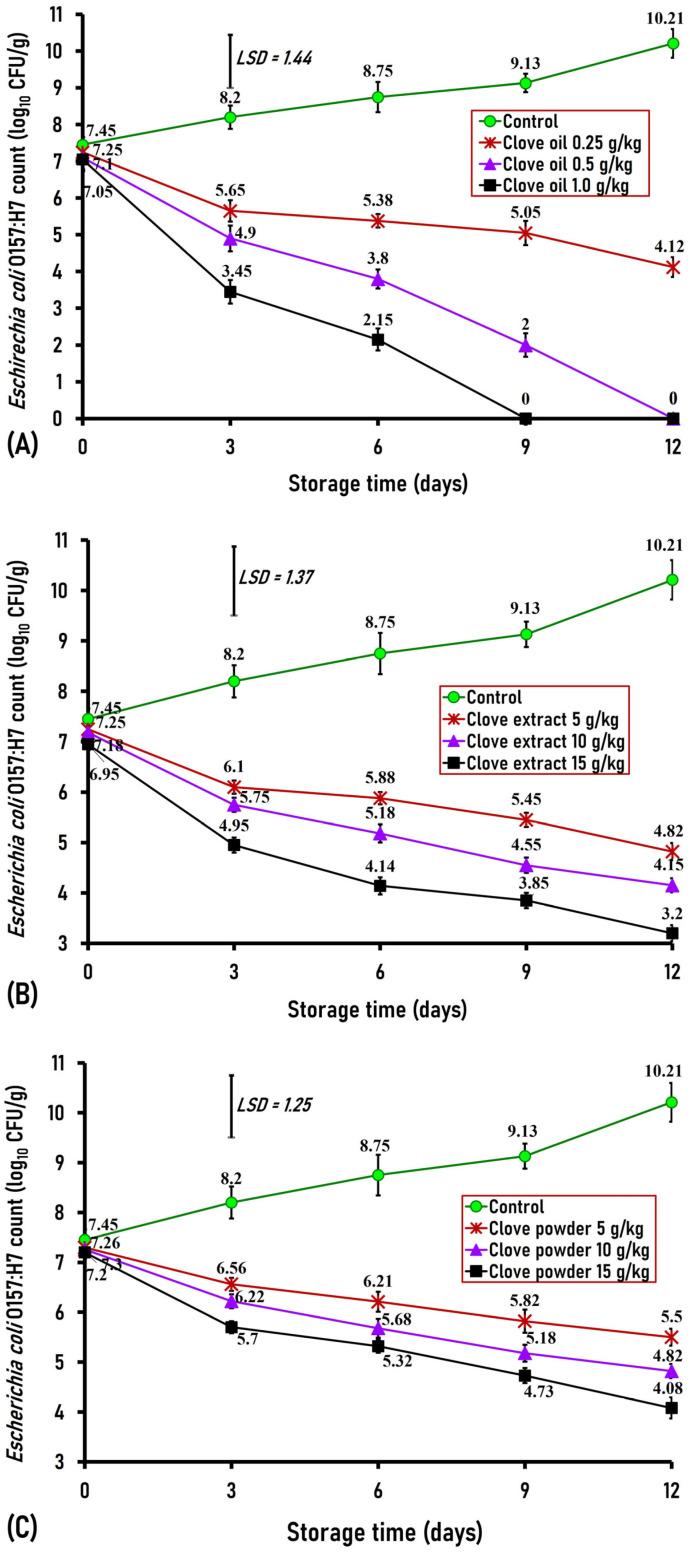
Antimicrobial effect of different concentrations of clove oil (**A**), extract (**B**), and powder (**C**) against *Escherichia coli* O157:H7 artificially inoculated into ground buffalo meat stored at 4 °C for 12 days. Data represent the mean ± SE values on each day of analysis. SE is represented by vertical bars; LSD is calculated at *p* < 0.01.

*Escherichia coli* reflects the hygienic standards of food and the potential for fecal contamination [5]. In the present study, the initial (day 0) *E*. *coli* O157:H7 counts inoculated in both control and clove-treated ground buffalo meat varied between 6.95 and 7.45 log_10_ CFU/g without a discernible difference between the control and the treated samples (Figure 8A–C). By day 3 of storage, ground buffalo meat that had been treated with clove oil (0.25, 0.5, and 1.0 g/kg), clove extract (0.5, 1.0, and 1.5 g/kg), and clove powder (2.5, 5, and 7.5 g/kg) showed a significant (*p <* 0.01) decline in the counts of *E*. *coli* O157:H7 by 2.55, 3.4, and 4.75 logs for clove oil-treated samples; 2.1, 2.45, and 3.25 logs for clove extract-treated samples; and 1.64, 1.98, and 2.5 logs for clove powder-treated samples when compared with the control, which contained 8.2 log_10_ CFU/g (Figure 8A–C). On the sixth day of storage, ground buffalo meat that had been treated with clove oil (0.25, 0.5, and 1.0 g/kg), clove extract (0.5, 1.0, and 1.5 g/kg), and clove powder (2.5, 5, and 7.5 g/kg) showed a significant (*p <* 0.05) decrease in *E. coli* O157:H7 counts by 3.37, 4.9, and 6.5 logs for clove oil-treated meat samples; 2.87, 3.5, and 4.6 logs for clove extract-treated samples; and 2.5, 3, and 3.5 logs for clove powder-treated samples when compared with the control, which had a count of 8.75 log_10_ CFU/g (Figure 8A–C). Interestingly, by the 9th day of storage and thereafter, ground buffalo meat containing clove oil at a level of 1.0 g/kg exhibited absolute inhibition in the growth of *E. coli* O157:H7, while the concentrations of 0.25 and 0.50 g/kg revealed significant (*p* < 0.01) declines in the count of *E. coli* O157:H7 by 4.08 and 7.13 logs, respectively, compared to the control, which contained a count of 9.13 log_10_ CFU/g. However, ground buffalo meat treated with clove extract (0.5, 1.0, and 1.5 g/kg) and clove powder (2.5, 5, and 7.5 g/kg) displayed a significant (*p <* 0.01) decline in the counts of *E*. *coli* O157:H7 by 3.6, 4.5, and 5.28 logs for clove extract-treated groups; and 3.31, 3.95, and 4.43 logs for clove powder-treated groups, respectively, when compared to the control sample, whose count was 9.13 log10 CFU/g (Figure 8A–C).

On the last storage day (day 12), ground buffalo meat in the control group had an elevated *E. coli* O157:H7 count of 10.21 log_10_ CFU/g. In contrast, ground buffalo meat treated with clove oil at 0.5 g/kg showed complete suppression of bacterial growth, while a treatment level of 0.25 g/kg resulted in a significant (*p* < 0.01) reduction in the *E*. *coli* count by 6.09 logs compared to the control’s 10.21 log_10_ CFU/g (Figure 6A). Additionally, adding clove extract at concentrations of 0.5, 1.0, and 1.5 g/kg caused significant (*p* < 0.01) decreases in *E*. *coli* O157:H7 counts by 5.41, 6.06, and 7.01 logs, respectively, relative to the control. Similarly, clove powder at 2.5, 5, and 7.5 g/kg led to notably lower *E*. *coli* O157:H7 counts by 4.71, 5.39, and 6.13 logs, respectively, compared to the control group (Figure 8A–C).

The present finding indicates the powerful antimicrobial effect of clove oil, clove extract, and clove powder as food additives against *E. coli* O157:H7, which is in agreement with that of Kuang et al. [31], who indicated that clove powder significantly reduced *E. coli* growth in vitro, and also with that of Zengin and Baysal [36], who revealed that the addition of clove oil resulted in growth inhibition of native coliforms artificially inoculated in ground beef.

The antimicrobial and sensory changes observed in clove-treated samples can be attributed to the bioactive compounds naturally present in clove, particularly eugenol, which is the principal phenolic component of clove oil and extract. Eugenol possesses strong antimicrobial activity through multiple mechanisms, including disruption of bacterial cell membranes, leakage of intracellular contents, inhibition of essential enzymes, and interference with cellular energy generation. These actions collectively reduce the viability of spoilage microorganisms and foodborne pathogens, which explains the significant log reductions observed in *S*. *aureus*, *S*. *enterica* serovar Typhimurium, and *E*. *coli* O157:H7 in the present study. However, little is known about how eugenol and other clove compounds function in complex meat matrices where the presence of fat, protein, and water may affect efficacy. These mechanisms, such as interactions with meat components, synergistic effects with other phenolics, and administration technique optimization to improve antibacterial efficacy while maintaining sensory quality, require further investigation.

## 4. Conclusions

This study provides one of the few comprehensive comparisons of clove oil, clove extract, and clove powder applied at multiple concentrations to buffalo meatballs. Clove oil and clove extract were the most effective treatments in slowing spoilage, reducing APCs and PCs by 1.5–2.6 logs, and extending refrigerated shelf life by 3–6 days compared with the control while maintaining acceptable sensory quality. In addition to improving shelf life, these treatments significantly reduced foodborne pathogens, producing 3–5.45 log reductions in *S*. *aureus* and *S*. *enterica* serovar Typhimurium and a 5.4-log reduction to complete elimination of *E*. *coli* O157:H7. Clove powder demonstrated antimicrobial activity but caused undesirable flavor and color changes, limiting its practical applicability. The novelty of this work lies in simultaneously comparing three clove forms at multiple doses in a single meat system, quantifying their differential effects on pathogens, spoilage flora, sensory quality, and shelf life. Although this study did not investigate the potential interactions of clove components with different processing conditions or packaging systems, it validates clove suitability in buffalo meat products and addresses an important regional and industrial need not fully covered by previous literature. Collectively, these findings highlight the dual benefit of clove oil and clove extract as natural preservatives that can be applied in the meat processing industry to enhance microbial safety, sustain sensory attributes, and prolong meat product shelf life.

## Figures and Tables

**Table 1:** Compositional analysis of clove essential oil, ethanolic clove extract, and clove powder based on the manufacturer-reported ranges.

Component	Clove Oil	Clove Extract	Clove Powder
Volatile Oil Content	70–90%	5–12%	12–18%
Eugenol	70–90%	35–60%	50–70% of oil
Eugenyl acetate	5–20%	1–8%	1–5%
β-Caryophyllene	5–12%	2–10%	5–10%
α-Humulene	0.5–3%	0.2–4%	1–3%
Caryophyllene oxide	0.3–1.5%	0.3–1.5%	0.3–1.0%
Tannins	Trace	5–20%	10–15%
Total Phenolics (as GAE)	<1%	150–350 mg GAE/g	10–15%
Total Flavonoids (as QE)	Trace	20–80 mg QE/g	1–2%
Crude Fiber	—	<5%	10–12%
Ash	—	<3%	5–7%
Carbohydrates	—	<5%	10–15%
Heavy Metals (Pb, Cd, As, Hg)	≤10 ppm	≤10 ppm	≤10 ppm
Microbial Load	<10^2^ CFU/g	<10^3^ CFU/g	<10^3^ CFU/g

**Table 2:** Mean values of the sensory evaluation score of ground buffalo meat treated with different concentrations of clove oil, extract, and powder during cold storage at 4 °C for 12 days.

Day of Storage	Sensory Characteristics		Control Ground Buffalo Meat	Clove Oil-Treated Ground Buffalo Meat			Clove Extract-Treated Ground Buffalo Meat			Clove Powder-Treated Ground Buffalo Meat		
				0.25 g/kg	0.5 g/kg	1.0 g/kg	0.5 g/kg	1.0 g/kg	1.5 g/kg	2.5 g/kg	5 g/kg	7.5 g/kg
Day 0	Clove flavor intensity ^x^		1.38 a	4.16 c	4.53 c	5.07 c	3.23 b	3.42 b	3.65 b	5.53 d	6.31 e	7.23 f
	Characteristic buffalo flavor ^x^		6.88 a	6.67 a	6.61 a	6.54 a	6.44 a	6.33 a	6.19 ab	5.66 b	5.42 b	5.23 b
	Tenderness ^x^	Initial	6.79 a	6.71 a	6.79 a	6.83 a	6.51 a	6.42 a	6.36 a	6.14 ab	5.82 ab	5.44 b
		Sustained	6.75 a	6.66 a	6.69 a	6.64 a	6.65 a	6.58 a	6.71 a	6.56 a	6.45 a	6.45 a
	Juiciness ^x^	Initial	6.79 a	6.75 a	6.65 a	6.66 a	6.65 a	6.61 a	6.52 a	6.15 ab	5.69 b	5.52 b
		Sustained	6.84 a	6.72 a	6.56 a	6.63 a	6.58 a	6.46 a	6.45 a	6.03 ab	5.58 b	5.37 b
	Off-flavor ^y^		1.15 a	1.21 a	1.18 a	1.21 a	1.22 a	1.25 a	1.33 a	1.3 a	1.2 a	1.5 a
	Overall acceptability ^z^		6.75 a	6.63 a	6.55 a	6.55 a	6.45 a	6.43 a	6.31 a	5.45 b	5.23 b	4.61 c
Day 3	Clove flavor intensity ^x^		1.35 a	4.05 c	4.23 c	4.77 c	3.03 b	3.12 b	3.35 b	5.37 d	6.01 d	6.82 d
	Characteristic buffalo flavor ^x^		6.63 a	6.51 a	6.45 a	6.43 a	6.43 a	6.39 a	6.27 ab	5.55 b	5.31 b	5.02 b
	Tenderness ^x^	Initial	6.69 a	6.62 a	6.69 a	6.65 a	6.49 a	6.35 a	6.29 a	6.09 ab	5.75 ab	5.39 b
		Sustained	6.58 a	6.52 a	6.59 a	6.65 a	6.39 a	6.25 a	6.21 a	6.02 ab	5.64 ab	5.27 b
	Juiciness ^x^	Initial	6.68 a	6.67 a	6.62 a	6.69 a	6.65 a	6.61 a	6.52 a	6.03 ab	5.49 b	5.32 b
		Sustained	6.58 a	6.57 a	6.60 a	6.65 a	6.58 a	6.52 a	6.43 a	5.83 ab	5.42 b	5.25 b
	Off-flavor y		1.18 a	1.25 a	1.19 a	1.21 a	1.41 a	1.35 a	1.42 a	1.42 a	1.52 a	1.75 a
	Overall acceptability ^z^		6.70 a	6.62 a	6.63 a	6.54 a	6.38 a	6.35 a	6.22 a	5.43 ab	5.24 b	4.60 c
Day 6	Clove flavor intensity ^x^		NE	3.78 a	4.09 ab	4.59 b	NE	3.05 c	3.24 c	NE	5.66 d	6.22 d
	Characteristic buffalo flavor ^x^			6.40 a	6.35 a	6.35 a		6.23 a	6.18 a		5.21 b	4.78 b
	Tenderness ^x^	Initial		6.49 a	6.55 a	6.55 a		6.19 a	6.15 a		5.68 ab	5.28 b
		Sustained		6.45 a	6.50 a	6.55 a		6.12 a	6.10 a		5.55 ab	5.18 b
	Juiciness ^x^	Initial		6.65 a	6.59 a	6.62 a		6.42 a	6.46 a		5.39 b	5.30 b
		Sustained		6.60 a	6.55 a	6.60 a		6.45 a	6.50 a		5.33 b	5.15 b
	Off-flavor ^y^			1.25 a	1.18 a	1.09 a		1.32 a	1.45 a		1.44 a	1.65 a
	Overall acceptability ^z^			6.55 a	6.58 a	6.68 a		6.35 a	6.24 a		5.18 b	4.44 c
Day 9	Clove flavor intensity ^x^		NE	NE	4.04 b	4.44 b	NE	NE	3.17 a	NE	NE	6.12 c
	Characteristic buffalo flavor ^x^				6.28 a	6.22 a			6.15 a			4.72 b
	Tenderness ^x^	Initial			6.42 a	6.51 a			6.10 a			5.21 b
		Sustained			6.45 a	6.49 a			6.05 a			5.18 b
	Juiciness ^x^	Initial			6.63 a	6.66 a			6.40 a			5.21 b
		Sustained			6.58 a	6.62 a			6.33 a			5.22 b
	Off-flavor ^y^				1.1 a	1.1 a			1.50 ab			1.72 b
	Overall acceptability ^y^				6.55 a	6.65 a			6.04 a			4.32 b
Day 12	Clove flavor intensity ^x^		NE	NE	NE	NE	NE	NE	NE	NE	NE	NE
	Characteristic buffalo flavor ^x^				6.12 a	6.14 a						
	Tenderness ^x^	Initial			6.29 a	6.58 a						
		Sustained			6.21 a	6.52 a						
	Juiciness ^x^	Initial			6.38 a	6.61 a						
		Sustained			6.41 a	6.58 a						
	Off-flavor ^y^				1.3 a	1.2 a						
	Overall acceptability ^z^				5.89 a	6.13 a						

^x^ The clove flavor intensity, ground buffalo flavor characteristic, tenderness, and juiciness were judged by an eight-point hedonic scoring scale from 1 (stands for extremely uncharacteristic beef flavor, tough, dry sample) to 8 (stands for extremely characteristic beef flavor, tender, juicy sample). ^y^ Off-flavor was determined by five-point hedonic scale values from 1 (denotes no off-flavor) to 5 (denotes extremely off-flavor). ^z^ The overall acceptability (overall mouthfeel) was determined by an eight-point hedonic scale with values from 1 (denotes extremely disliked) to 8 (denotes extremely liked). Mean values with a different letters in the same row for all treatments are significantly different (*p* < 0.05). NE: not evaluated as a result of visible deteriorative changes.

## Data Availability

The original contributions presented in this study are included in the article/Supplementary Materials. Further inquiries can be directed to the corresponding authors.

## Supplementary Materials

The following supporting information can be downloaded at: https://www.mdpi.com/article/10.3390/foods15010113/s1, Table S1. The Beef Sensory Evaluation Form.

## References

[1] Sung S.Y., Sin L.T., Tee T.T., Bee S.T., Rahmat A.R., Rahman W.A.W.A., Tan A.-C., Vikhraman M. (2013). Antimicrobial agents for food packaging applications. Trends Food Sci. Technol..

[2] Ahmed S.A., Sarangi S.K. (2013). Analysis of bacterial contamination in fresh and finished meat products and their molecular identification. Int. J. Pharm. Sci. Invent..

[3] Hait J., Bennett R., Lampel K.A., Al-Khaldi S., Cahill S.M. (2012). *Staphylococcus* *aureus*. Bad Bug Book, Foodborne Pathogenic Microorganisms and Natural Toxins.

[4] Hait J., Tallent S., Melka D., Keys C., Bennett R. (2012). *Staphylococcus aureus* Outbreak Investigation of an Illinois Bakery. J. Food Saf..

[5] Synge B.A. (2000). Verocytotoxin-producing *Escherichia coli*: A veterinary view. Symp. Ser. Soc. Appl. Microbiol..

[6] Jaybhaye A., Deb M. (2021). Pathogenesis of *Escherichia coli*: A Clinical Findings. J. Pharm. Res. Int..

[7] Carocho M., Morales P., Ferreira I.C. (2015). Natural food additives: Quo vadis?. Trends Food Sci. Technol..

[8] Cortes-Rojas D.F., de Souza C.R., Oliveira W.P. (2014). Clove (*Syzygium aromaticum*): A precious spice. Asian Pac. J. Trop. Biomed..

[9] El-Maati M.F.A., Mahgoubb S.A., Labiba S.M., Al-Gabya A.M.A., Ramadan M.F. (2016). Phenolic extracts of clove (*Syzygium aromaticum*) with novel antioxidant and antibacterial activities. Eur. J. Integr. Med..

[10] Lee K.G., Shibamoto T. (2001). Antioxidant property of aroma extract isolated from clove buds [*Syzygium aromaticum* (L.) Merr. et Perry]. Food Chem..

[11] Bakkali F., Averbeck S., Averbeck D., Idaomar M. (2008). Biological effects of essential oils. Food Chem. Toxicol..

[12] Nunez L., Aquino M.D. (2012). Microbicide activity of clove essential oil (*Eugenia caryophyllata*). Braz. J. Microbiol..

[13] Xu J.G., Liu T., Hu Q.P., Cao X.M. (2016). Chemical composition, antibacterial properties and mechanism of action of essential oil from clove buds against *Staphylococcus aureus*. Molecules.

[14] Shan B., Cai Y.Z., Sun M., Corke H. (2005). Antioxidant capacity of 26 spice extracts and characterization of their phenolic constituents. J. Agric. Food Chem..

[15] Sallam K.I., Ishioroshi M., Samejima K. (2004). Antioxidant and antimicrobial effects of garlic in chicken sausage. LWT.

[16] (2013). Microbiology of the Food Chain—Horizontal Method for the Enumeration of Microorganisms. Part 1: Colony count at 30 °C by the pour plate technique. https://www.iso.org/standard/53728.html.

[17] (2019). Microbiology of the Food Chain—Horizontal Method for the Enumeration of Psychrotrophic Microorganisms. https://www.iso.org/standard/67437.html.

[18] Rahn K., De Grandis S.A., Clarke R.C., McEwen S.A., Galan J.E., Ginocchio C., Curtiss R., Gyles C.L. (1992). Amplification of an *invA* gene sequence of *Salmonella typhimurium* by polymerase chain reaction as a specific method of detection of *Salmonella*. Mol. Cell. Probes.

[19] Sallam K.I., Abd-Elghany S.M., Elhadidy M., Tamura T. (2015). Molecular characterization and antimicrobial resistance profile of methicillin-resistant *Staphylococcus aureus* in retail chicken. J. Food Prot..

[20] Sallam K.I., Mohammed M.A., Ahdy A.M., Tamura T. (2013). Prevalence, genetic characterization and virulence genes of sorbitol-fermenting *Escherichia coli* O157: H-and *E*. *coli* O157: H7 isolated from retail beef. Int. J. Food Microbiol..

[21] Sharma H., Mendiratta S.K., Agarwal R., Goswami M. (2019). Optimization of various essential oils and their effect on the microbial and sensory attributes of chicken sausages. Agric. Res..

[22] Zhang H., Peng X., Li X., Wu J., Guo X. (2017). The application of clove extract protects Chinese-style sausages against oxidation and quality deterioration. Korean J. Food Sci. Anim. Resour..

[23] Ahmed B.H., Mohammed N.A.M. (2024). Effects of Adding Clove (*Syzygium aromaticum* L.) Seed Extract on Oxidative Stability, Microbial Activity, and Sensory Attributes in Beef Patties During Refrigerated Storage. Kurd. J. Appl. Res..

[24] Jay J.M. (2002). A review of aerobic and psychrotrophic plate count procedures for fresh meat and poultry products. J. Food Prot..

[25] International Commission on Microbiological Specifications for Foods (ICMSF) (1986). Microorganisms in Foods. 2. Sampling for Microbiological Analysis: Principles and Specific Applications.

[26] Salehi B., Zakaria Z.A., Gyawali R., Ibrahim S.A., Rajkovic J., Shinwari Z.K., Khan T., Sharifi-Rad J., Ozleyen A., Turkdonmez E. (2019). Piper species: A comprehensive review on their phytochemistry, biological activities and applications. Molecules.

[27] Sharma H., Mendiratta S.K., Agarwal R.K., Kumar S., Soni A. (2017). Evaluation of anti-oxidant and anti-microbial activity of various essential oils in fresh chicken sausages. J. Food Sci. Technol..

[28] Ali F., Abdel-Atty N., Helmy E. (2018). Improving the quality and extending the shelf life of chilled fresh sausages using natural additives and their extracts. J. Microbiol. Biotechnol. Food Sci..

[29] Omuero R.O., Tanny P.C., Chikwem U.J., Chikwem N.J., Chikwem J.O. (2017). Determination of the antimicrobial effect of cloves for extending the shelf life of chicken meat. IHE Lincoln Univ. J. Sci..

[30] Holzapfel W.H., Davies A., Board R. (1998). The gram-positive bacteria associated with meat and meat products. The Microbiology of Meat and Poultry.

[31] Jirovetz L., Buchbauer G., Stoilova I., Stoyanova A., Krastanov A., Schmidt E. (2006). Chemical Composition and Antioxidant Properties of Clove Leaf Essential Oil. J. Agric. Food Chem..

[32] Vieira B.B., de Carvalho E.A., da Rocha Bispo A.S., Ferreira M.A., Evangelista-Barreto N.S. (2020). Efficiency of chitosan synergism with clove essential oil in the coating of intentionally contaminated Tambaqui fillets. Semin. Ciências Agrárias.

[33] Tajik H., Farhangfar A., Moradi M., Razavi Rohani S.M. (2014). Effectiveness of clove essential oil and grape seed extract combination on microbial and lipid oxidation characteristics of raw buffalo patty during storage at abuse refrigeration temperature. J. Food Process. Preserv..

[34] Kuang X., Li B., Kuang R., Zheng X., Zhu B.O., Xu B., Ma M. (2011). Granularity and antibacterial activities of ultra-fine cinnamon and clove powders. J. Food Saf..

[35] Keskin D., Toroglu S. (2011). Studies on antimicrobial activities of solvent extracts of different spices. J. Environ. Biol..

[36] Nassan M., Mohamed E., Abdelhafez S., Ismail T. (2015). Effect of clove and cinnamon extracts on experimental model of acute hematogenous pyelonephritis in albino rats: Immunopathological and antimicrobial study. Int. J. Immunopathol. Pharmacol..

[37] Shan B., Cai Y.Z., Brooks J.D., Corke H. (2007). The in vitro antibacterial activity of dietary spice and medicinal herb extracts. Int. J. Food Microbiol..

[38] Billah M.M., Rahman M.S. (2024). *Salmonella* in the environment: A review on ecology; antimicrobial resistance, seafood contaminations, and human health implications. J. Hazard Mater. Adv..

[39] Zengin H., Baysal A.H. (2015). Antioxidant and antimicrobial activities of thyme and clove essential oils and application in minced beef. J. Food Process. Preserv..

[40] Angienda P.O., Onyango D.M., Hill D.J. (2010). Potential application of plant essential oils at sub-lethal concentrations under extrinsic conditions that enhance their antimicrobial effectiveness against pathogenic bacteria. Afr. J. Microbiol. Res..

[41] Shan B., Cai Y.Z., Brooks J.D., Corke H. (2009). Antibacterial and antioxidant effects of five spice and herb extracts as natural preservatives of raw pork. J. Sci. Food Agric..

