# Supplementary material for: Antimicrobial Effect of Clove Against Foodborne Pathogens in Ground Buffalo Meat During Refrigerated Storage
Source: Foods. 2025 Dec 31;15(1):113. doi: 10.3390/foods15010113 (PMC12785366; doi:10.3390/foods15010113)
Supplement: Supplementary file 1 [file foods-15-00113-s001.zip › foods-4016589-supplementary.pdf]

***Table S1**. The Beef Sensory Evaluation Form

Panelist ID-------------------- Date----------------------- Time------------------ am/pm Project--------------------

Sample	JUICINESS		TENDERNESS		FLAVOR INTENSITY	BEEF FLAVOR	OVERALL MOUTHFEEL	OFF FLAVOR
	INITIAL	SUSTAINED	INITIAL	SUSTAINED				
1								
2								
3								
4								
5								
6								
7								
8								
9								
10								
11								
12								

**JUICINESS**	**TENDERNESS**	**FLAVOR INTENSITY**
8 Extremely juicy	8 Extremely tender	8 Extremely intense
7 Very juicy	7 Very tender	7 Very intense
6 Moderately juicy	6 Moderately tender	6 Moderately intense
5 Slightly juicy	5 Slightly tender	5 Slightly intense
4 Slightly dry	4 Slightly tough	4 Slightly bland
3 Moderately dry	3 Moderately tough	3 Moderately bland
2 Very dry	Very tough	2 Very bland
1 Extremely dry	1 Extremely tough	1 Extremely bland

**BEEF FLAVOR**	**OVERALL BEEF MOUTHFEEL**	**OFF-FLAVOR**
8 Extremely characteristic beef flavor	8 Extremely beef-like mouthfeel	5 Extremely off-flavor
7 Very characteristic beef flavor	7 Very beef-like mouthfeel	4 Very off-flavor
6 Moderately characteristic beef flavor	6 Moderately beef-like mouthfeel	3 Moderate off-flavor
5 Slightly characteristic beef flavor	5 Slightly beef-like mouthfeel	2 Slight off-flavor
4 Slightly uncharacteristic beef flavor	4 Slightly non-beef-like mouthfeel	1 None
3 Moderately uncharacteristic beef flavor	3 Moderately non-beef-like mouthfeel	
2 Very uncharacteristic beef flavor	2 Very non-beef-like mouthfeel	
1 Extremely uncharacteristic beef flavor	1 Extremely non-beef-like mouthfeel	

*Beef Sensory Evaluation Form (Texas Tech University)
